# An Investigation into the Effect of Body Condition and Other Gilt Characteristics on Estrus Responses Post Altrenogest Treatment and on Reproductive Performance

**DOI:** 10.3390/ani15050623

**Published:** 2025-02-20

**Authors:** Sandra Krause, Nina Bauske, Haukur L. Sigmarsson, Alexander Grahofer, Hendrik Tietje, Daniel Sperling, Johannes Kauffold

**Affiliations:** 1 Clinic for Ruminants and Swine, Faculty of Veterinary Medicine, University of Leipzig, An den Tierkliniken 11, 04103 Leipzig, Germany; 2 Clinic for Swine, Department of Clinical Veterinary Science, Vetsuisse Faculty, University of Bern, 3012 Bern, Switzerland; alexander.grahofer@unibe.ch; 3 Statistical Office of Hamburg and Schleswig-Holstein, Fröbelstraße 15-17, 24113 Kiel, Germany; 4 Ceva Santé Animale, 10 Avenue de la Ballastière, 33500 Libourne, France; daniel.sperling@ceva.com

**Keywords:** gilts, altrenogest, body condition, puberty, reproduction

## Abstract

This study was conducted to investigate the effect of body condition (BC) and other characteristics on estrus responses post altrenogest treatment and on reproductive performance in 161 gilts, bred artificially, treated with 20 mg altrenogest orally for 18 consecutive days. Parameters recorded at the beginning of altrenogest treatment included age and body weight. Backfat (BFT) and muscle thickness (MT), both defining BC, puberty status and uterine size (UtS, based on cross-sectional areas of the uterine horns) were recorded by real-time-ultrasound (RTU), and BFT, along with MT together with ovary status, were recorded again at the end of altrenogest treatment. Estrus rate (ER), interval of last altrenogest treatment onset of estrus (ATOE), conception (CR) and farrowing (FR) rates, litter size characteristics (total, life, and still born piglets; TB, LB, SB) and individual piglet birth weights (IPWs) were recorded. Average age and weight were 252.6 ± 7.5 days and 153.0 ± 12.4 kg, respectively. BFT and MT were 0.9 ± 0.2 and 6.0 ± 0.4 cm, respectively, at the beginning of altrenogest treatment, and similar at the end. 147 gilts were determined as pubertal (PUB) and 14 prepubertal (PRE). All gilts only had follicles of 3.2 ± 0.4 mm on average at the end of altrenogest treatment. ER, ATOE, CR, FR, TB, LB, SB and IPW were 99.4%, 5.9 ± 0.5 days, 100%, 95.6%, 18.1 ± 2.6, 17.4 ± 2.5, 0.7 ± 1.0, 1.3 ± 0.2 kg, respectively. PUB and PRE did not differ, or only marginally differed, in all recorded parameters. BC or pubertal status did not have a great impact on the efficacy of altrenogest treatment or reproductive performance. However, UtS was significantly smaller in PRE than in PUB (0.51 ± 0.15 cm^2^ and 1.63 ± 0.48 cm^2^, respectively). There was a very slight significant relationship between UtS and LB (r = 0.19, *p* = 0.03) and a tendency towards TB (r = 0.15, *p* = 0.09). In conclusion, under the conditions of this study, BC, age and pubertal status did not have an effect on the efficacy of altrenogest or the reproductive performance of hyper-prolific gilts of Danish genetic origin, but uterine size may have had a positive effect.

## Simple Summary

Gilts have to be in optimal body condition and sexually mature before first mating. However, with hyper-prolific breeds, gilts grow faster and become leaner. Increasing leanness may put new challenges on gilt management, with respect also to their responses to lipophilic drugs, like altrenogest, used to synchronize estrus. However, under the conditions of this study, neither body condition (based on backfat and muscle thickness) or age, nor body weight, nor pubertal status and uterine size had a substantial effect on the efficacy of altrenogest or on the reproductive performance of Danish genetic gilts. This may be due to the fact that the tested gilt population was relatively homogenous in this regard, except for uterine size.

## 1. Introduction

Good gilt replacement management is crucial to swine production [1,2,3]. Patterson et al. [4] have defined 10 key principles for improved sow lifetime performance. These principles include early age at puberty by day 200, in order to breed gilts at their second (or later) estrus (which would be between days 220 and 240) at acceptable target weights of 135–160 kg. Backfat and muscle thickness should also be measured, summarized in the following as body condition. Measuring both thicknesses with ultrasound at the P2 location, as suggested by Maes et al. [5], offers a more precise evaluation of body condition compared to visual body scoring. Recommended muscle thicknesses for gilts at certain ages are not yet available to the authors’ knowledge. Optimum backfat thickness is considered to be between 13 and 15 mm [3,6]. On the one hand, gilts should not have too little backfat, as this stores, e.g., the hormone leptin, which has important effects on reproduction processes [7,8]. On the other hand, gilts should not be too fat, and therefore too heavy, as this can lead to, e.g., birth difficulties [2,4]. However, target weight and at least one heat-no-service, as well as acclimatization to the herd health status, seem to be more important than backfat and age [9,10]. In practice, a random gilt pool does not usually fulfill all the requirements [11,12]. For example in the study of Díaz et al. [12], the gilts’ body weight at breeding ranged from 113 to 192 kg and backfat thickness from 14 to 33 mm. With the use of modern hyper-prolific breeds, gilts have also become leaner and faster growing, which brings new challenges to the gilt replacement management in order to ensure a constant flow of high quality gilts into the breeding herd [3,13].

These challenges, particularly leanness, may affect responsiveness to lipophilic drugs. such as the orally active progestin altrenogest, which suppresses LH and is used to synchronize gilts [14,15,16], and is commonly fed to gilts at 15–20 mg/day for 14–18 days (there are different protocols for the synchronization of altrenogest-based products registered in EU countries and in the US) [16,17,18]. Thitachot et al. [19], for instance, observed an effect of backfat thickness on the interval from altrenogest withdrawal to estrus in Landrace × Yorkshire gilts, a breed that, according to its litter traits (i.e., <12 total born piglets in the study of Thitachot et al. [19]), would be considered “conventional”. The less backfat (i.e., <13.5 mm), the shorter this interval. These authors suggested that adipose tissue serves as a reservoir for altrenogest; the more fat, the bigger the storage of altrenogest, as has been also proven for progesterone [20]. Altrenogest may still be released after withdrawal from adipose tissue in fatter gilts, leading to a prolongation of its effect on LH suppression and, subsequently, to a delayed onset of estrus [19].

As highlighted by Bortolozzo et al. [3] that “hyper-prolific breeds are leaner”, and based on the results of the study of Thitachot et al. [19] showing an effect of backfat thickness on estrus post altrenogest in a more conventional breed, this investigation was conducted to test the effect of body condition (based on backfat and muscle thickness) on estrus response post altrenogest treatment and on reproductive performance in gilts of hyper-prolific Danish genetic. Other parameters tested included age and body weight, puberty status, and ultra sonographically determined uterine size.

## 2. Materials and Methods

### 2.1. Farm Characteristics and General Management

The study was performed on a farrow–wean swine farm in Saxony, Germany, at coordinates 50°44′42.0” N, 12°18′26.5” E. At the time the study was conducted, outside temperature ranged from −2.3 °C to 19.6 °C during the day and between −1.2 °C and 18.7 °C at night. Humidity ranged from 47.9% to 91.9%. The farm had a total inventory of 2200 productive sows, with own gilt replacement. The genetic background was purebred Yorkshire (a nucleus of approximately 200 sows) and the remainder were hybrid Yorkshire (♀) × Danish Landrace (♂). A weekly batch farrowing system with four weeks lactation was employed (average lactation length was approximately 25 days). According to data records of the year preceding the study, the farrowing rate was between 89.6% and 93.1%. Total and live born piglets were 18.7 and 17.5, respectively, resulting in a total of 42.0 pigs born alive/sow/year and 39.8 pigs weaned/sow/year. The farm was specifically pathogen-free of all reportable and common diseases in Germany. Routine mass treatment procedures of gilts and sows included vaccination against PPV, SIV, Mycoplasma, PCV2, *E. coli* and Clostridia (*C. perfringens* type A, C), as well as deworming.

### 2.2. General Gilt Management

Piglet birth weight was periodically recorded and, according to producer information, generally reported to be above 1 kg. Average wean weight of pigs, including future replacement gilts, was approximately 6.1 kg. At weaning, piglets were moved into the nursery, where they were kept in groups (n = 66; space allowance: 0.2 m^2^/weaner) on slatted floors until a weight of 30 kg at approximately 11 weeks of age. In nursery, dry nursery diet (13.8 MJ ME/kg) was delivered in troughs and water through nipple drinkers, both ad libitum. At 11 weeks of age, gilts were moved into the gilt developer unit (GDU), where they were again kept in groups with 20 gilts/group (space allowance: 0.75 m^2^/gilt until a body weight of 90 kg; 1.0 m^2^/gilt thereafter). Gilts weighing 30–90 kg were fed a dry diet designed for gilts (11.2 MJ/kg) ad libitum and then switched to liquid feed as a combination of early gestational and lactational feed (11.7 MJ ME/kg). Gilts remained in the GDU until an age of approximately 245 days at a weight of approximately 140 kg. Thereafter, gilts were transferred to a second GDU, where they stayed until 273 days of age, weighing approximately 150 kg, then transferred to the sow unit for breeding. An extra 200 g of dextrose (as topdressing) was given orally over five days prior to breeding for purposes of flushing. At none of the production stages, neither boar contact occurred nor estrus signs were recorded, i.e., it was not known to the farm personnel and was not considered in daily management whether gilts were pubertal or prepubertal at breeding (based on the fact that overall reproductive performance of gilts continued to be high over the years).

### 2.3. Experimental Design

#### 2.3.1. Gilts Included, Altrenogest Application and Breeding Protocol

A total of 166 gilts in 7 batches (22–24 gilts/batch) were enrolled. Ten gilts were purebred Yorkshire (YS) and 156 hybrid Yorkshire (♀) × Danish Landrace (♂) gilts (YS–DL) (random breed distribution). Gilts were trained to uptake altrenogest by drenching, using apple juice, for three consecutive days prior to first altrenogest treatment. Gilts were put on altrenogest (Altresyn ^®^, Ceva Tiergesundheit, Düsseldorf, Germany) according to the leaflet information, i.e., 20 mg (5 mL) per gilt per day orally through drenching, always at 07.30 AM, for 18 consecutive days. No other hormones were administered. Altrenogest treatment started at approximately 255 days of age, when gilts were still confined in groups in the second GDU. Approximately 1 h after last altrenogest application, gilts were transferred into the sow unit. Boar contact started the day after last altrenogest treatment and was performed twice a day in order to stimulate and recognize heat (AM/PM), and heat checking results were recorded. Gilts were artificially inseminated (cervical AI) based on a positive standing response (a reflex during the estrus, where the gilt stands still with her back arched, triggered by boar contact, the pressure of the insemination catheter, or a human’s knee or back pressure, which indicates that the gilt would tolerate the boar’s jump [21]), also on an AM/PM basis (i.e., gilts in heat AM were bred PM, and those in heat PM were bred AM), using purchased extended liquid semen. Average number of AIs was 3, with a range of 2–4 per gilt.

#### 2.3.2. Parameters Recorded at the Beginning of Altrenogest Treatment

**Figure 1:**
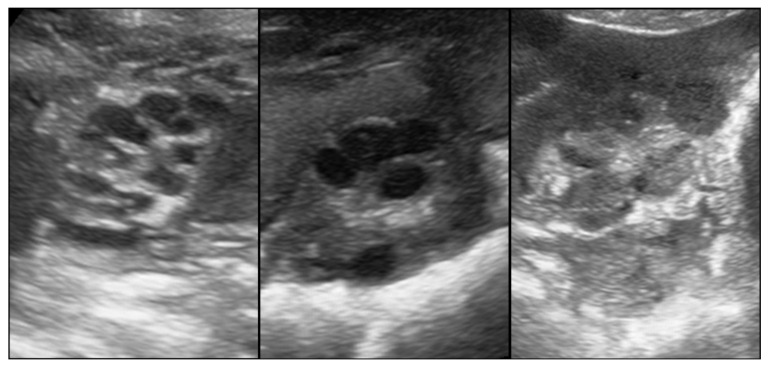
(**left**): An ovary with small follicles indicative of a prepubertal gilt. (**middle**) and (**right**): An ovary with large follicles, as well as Corpora lutea, respectively, indicative of a pubertal gilt.

**Figure 2:**
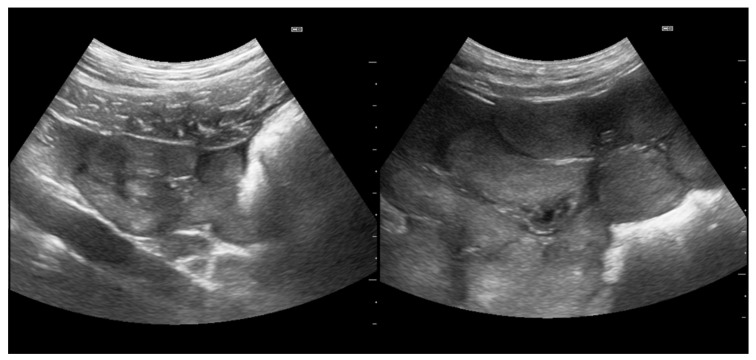
(**left**): Cross-sections of uterine horns of a prepubertal gilt measuring less than 1 cm in diameter. (**right**): Cross-sections of uterine horns of a pubertal gilt measuring more than 1.5 cm in diameter. Scale bar on left margins in 0.5 cm increments.

Parameters recorded included the following: 1. pubertal status: Transabdominal Real-time Ultrasonography (RTU) using a Fazone CB (Fujifilm, Tokyo, Japan) and a convex probe at 5 MHz (3–9 MHz; FZT C9-3) was performed at 6–8 cm penetration depth, closely following the procedure, as reported previously [22], the determination based on ovary (Figure 1) and uterus (Figure 2) assessment. Gilts having an ovary with only small follicles (between 2 and 4 mm in size) and/or via a subjectively assessed cross-sectional area of uterine horns of ≤1.0 cm^2^ (equal approximately too ≤ 1.0 cm diameter) were considered prepubertal. If the ovaries had larger (pre-ovulatory) follicles, Corpora haemorrhagica or Corpora lutea (Figure 1), and/or cross-sections of the uterine horns ≥ 1.2 cm^2^ (equal approximately to ≤ 1.2 cm diameter), the gilt was judged as pubertal. 2. Individual age. 3. Body weight (using a portable scale (Meier-Brakenberg GmbH and Co. KG, Brakenberg, Germany). 4. Backfat thickness (BFT). 5. muscle thickness (MT; *Musculus longissimus dorsi*). Measurements for BFT and MT were carried out by RTU using the aforementioned equipment at the P2 location and a penetration depth of 5 cm following the procedure described by Maes et al. [5], with some modifications. In brief, scanning was performed on both body sides at the last rip 5–6 cm lateral to the spine bone. Both measurements, i.e., left and right, were then averaged to give the mean BFT and MT, respectively, for an individual gilt. BFT and MT were used to define the body condition of each gilt. 6. Uterine size: defined as mean cross-sectional area of the uterine horn and determined based on recorded video sequences that were retrospectively analyzed using the computer program MicroDicom © viewer (Version 3.2.2 [Build 831] 32 bit, Sofia, Bulgaria). A total of 132 pubertal and 13 prepubertal gilts were successfully measured.

#### 2.3.3. Parameters Recorded at the End of Altrenogest Treatment

The ovary was monitored as aforementioned in order to assess the effect of altrenogest, which was considered “successful” if only small to midsize follicles were present (i.e., no Corpora lutea or Corpora haemorrhagica).

#### 2.3.4. Parameters Recorded for Reproductive Performance

The estrus rate after last altrenogest treatment as well as the interval between last altrenogest treatment and onset of estrus was determined. Pregnancy was checked by farm personnel twice between days 27 and 42 after last AI by RTU using a portable ultrasound unit (Multiscan Class II Typ B, MS Schippers GmbH, Kerken, Germany), and the conception rate was calculated (as the percentage pregnant/bred gilts). Farrowing rate (as the percentage of farrowed/bred gilts), as well as litter characteristics (i.e., total born, born alive and stillborn piglets), were recorded. Individual and total litter weight of selected litters (n = 70) were recorded using the portable scale Typ 66172 (SOEHNLE, Nassau, Germany).

### 2.4. Statistical Analysis

Statistical analysis was carried out in SAS Version 9.4 (SAS Institute Inc., Cary, NC, USA). From 166 gilts initially included, five were excluded due to locomotion problems post puberty check, and 161 gilts remained for analysis. Descriptive statistics were used to present the overall data set. It was decided to employ the Kruskal–Wallis test (as a nonparametric test that can be independently used regardless of the distribution of data) to compare most parameters within the overall data set, as well as between pubertal and prepubertal gilts. For comparison of the distribution of gilts according to the interval between last altrenogest treatment and the onset of estrus, as well as for conception and farrowing rates between pubertal and prepubertal gilts, the Chi–quadrat test was used. Fisher’s exact test was used due to expected frequencies below 5 in some contingency tables (e.g., distribution of gilts according to the interval between last altrenogest treatment and the onset of estrus), where the Chi-square test would not be appropriate. For the analysis of the relationship of data, the Pearson correlation was employed. A *p*-value of ≤0.05 was considered significant.

## 3. Results

### 3.1. All Gilts (Overall Data)

Descriptive statistics for all data included in the analysis is presented in Table 1. From 166 gilts initially included, five were excluded due to locomotion problems post puberty check, and 161 gilts remained for analysis. Almost all (except one) gilts were in estrus 5.9 ± 0.5 days post altrenogest, on average, and bred. All gilts had small to midsize follicles at the end of altrenogest treatment (including the gilt that failed to show estrus). Backfat and muscle thickness increased slightly (although statistically non-significant) if compared between the beginning and the end of altrenogest treatment. Overall production was high. All gilts conceived, and 153 farrowed. Of the seven that did not farrow, four aborted and three returned to estrus after positive pregnancy check. Both the overall total and the number of live born piglets was high, while the number of stillborn piglets was low. Individual piglet weight averaged 1.3 ± 0.2 kg.

Almost all, i.e., 156 of the 160 gilts with estrus after last altrenogest treatment, were observed in estrus within 6.5 days (and within 1.5 days, i.e., between days 5 and 6.5), with a majority of almost 50% on day 6 (Table 2; *p* ≤ 0.05).

There were not many relationships between body condition (i.e., BFT and MT), age, body weight, and follicle size at the end of altrenogest treatment, nor between production traits (Table 3) The few significant relationships were only slight and sometimes confounding. Age, e.g., had a positive effect on follicle size at the end of altrenogest treatment. However, while MT at the beginning of altrenogest treatment had a positive effect on the interval between last altrenogest treatment and the onset of estrus, it negatively correlated with conception and farrowing rate.

### 3.2. Pubertal Versus Prepubertal Gilts

A clear majority of gilts (n = 147) was determined as pubertal and only a few gilts as prepubertal (n = 14). There were only a few statistically proven differences between prepubertal and pubertal gilts, which included MT at the beginning and follicle size at the end of altrenogest treatment (Table 4). The interval between the end of altrenogest treatment and the onset of estrus was statistically not significant, as were all results related to reproductive performance. However, mean uterine size of pubertal gilts was much higher than of prepubertal gilts.

### 3.3. Relationship Between Uterine Size and Production Traits

Uterine size did not have an effect on conception or pregnancy rate, as well as on the number of stillborn piglets and mean piglet weight. However, there was a significant positive effect on the number of live born piglets (r = 0.19, *p* = 0.03), as well as on the number of total born piglets (albeit only in tendency; r = 0.15, *p* = 0.09).

## 4. Discussion

This study was conducted to investigate the effect of body condition, age and body weight, as well as puberty status, on the efficiency of altrenogest treatment and subsequent reproductive performance in gilts.

The farm where the study was conducted had a record of high production of gilts in the period preceding this study (e.g., between 1 November 2018 and 1 November 2019, a mean conception rate of 95.6%, a mean farrowing rate of 92.7%, and a mean litter size of 19.1 piglets). This was again confirmed in this investigation with almost 100% conception and a slightly smaller farrowing rate, as well as an extraordinary high litter size, and is certainly related to highly efficient reproduction management together with the use of gilts of the hyper-prolific Danish genetic stock.

Gilt management in this study was unusual. Firstly, boar exposure, known to be one key factor for early puberty attainment (i.e., <200 days), was not performed at any stage of the gilt’s life except during the time before breeding. Nevertheless, only 8.7% of the gilts were still prepubertal at around 250 days, which seems reasonable in number compared to the 34.3% in the recent Spanish study of Vela et al. [23] using Topigs TN70 gilts at 240 days of age, also without boar contact. It seems that the settings and management practices on the study farm (even without boar contact) did not negatively affect reproduction outcomes. Secondly, gilts were bred at approximately 270 days and at a projected body weight of 165–170 kg. This age is not the optimum for gilts at first breeding, reported to be 220–240 days, but the weight is only a little over the target of 135–160 kg [3,24,25]. According to Bortolozzo et al. [3], age (but also backfat) is not as important as is target body weight, given that breeding is carried out in the second or later estrus and the state of health is provided (“adapted to herd health”). Most gilts were bred in their second or later estrus and the herd was specific pathogen free, which may explain the high performance of the gilts, at least in their first parity (which was recorded). Overweight and bred at an older age have been associated with reduced productivity and reduced longevity [2,10,26,27,28], but were not recorded in this study.

Muscle and backfat thicknesses were recorded to determine body condition of the gilts. Both thicknesses slightly increased during the interval between beginning and end of altrenogest treatment. Prepubertal and pubertal gilts did not differ in either thickness. Whether muscle tissue is directly linked to reproduction in gilts is not known, nor are there recommended muscle thicknesses for gilts at certain ages available, to the author’s knowledge. That muscle is important for reproduction comes from, e.g., studies in lactating sows, where those losing muscle to below threshold levels during lactation display impaired fertility (lower conception, as well as lower birth weights, and higher within-litter variability in birth weights [29]). In the studies of Gaughan et al. [30], the fat deposition and protein deposition were higher (*p* < 0.05) in those gilts that generally attained puberty but the authors concluded that factors which restrict growth and maturity are more important than the effect of leanness on reproductive development. Age at puberty was also not affected by diet or overall lean growth at stimulation, confirming that innate variability in sexual development of commercial genotypes determines onset of sexual maturity, rather than growth performance [31]. Backfat thicknesses in this study were 11 and 13 mm, respectively, for pubertal and prepubertal gilts, which is within the range, or close to the 13–15 mm that is recommended at first breeding [3,6,19]. Fat tissue is known to play a pivotal role in reproduction processes (e.g., through the effects of leptin), and a threshold amount of backfat is thus necessary for optimum reproduction [7,8].

All gilts were shown to have only small to midsize follicles at the end of an 18-day altrenogest treatment with 20 mg (5 mL) per day per gilt. Altrenogest apparently blocked follicular growth and prevented ovulation successfully, while Corpora lutea were able to regress spontaneously [14,15,16,32], reviewed by Kraeling et al. [1]. The fact that altrenogest was also “effective” in prepubertal gilts in this study is not surprising, as altrenogest suppresses LH, which is already naturally suppressed in prepubertal gilts [1,16,18,33]. In this study, almost all gilts were observed in estrus by day 6.5 after last altrenogest treatment, and very synchronous within 1.5 days, which allowed very synchronous breeding in their respective batches. Both the numbers of gilts in estrus and estrus synchrony were slightly higher than in the study of Martinat-Botté et al. [16], where 94% of gilts were observed in estrus 4–8 days post altrenogest withdrawal, and much better than in the study of Dimitrov et al. [34], with an estrus rate of 86.99%, where the interval from last day of treatment to estrus was 3–10 days, while most (67.31%) exhibited estrus on day 4 and 5. One reason for the better results of this study compared to the aforementioned might be related to the comparatively low and uniform backfat thicknesses of the gilts, as Thitachot et al. [19] have shown a positive correlation between backfat thickness and the interval between altrenogest withdrawal and estrus occurrence. Interestingly, almost all prepubertal animals showed estrus post altrenogest in this study, indicating that they had been sufficiently matured to attain puberty.

None of the observed parameters recorded in this study with respect to body condition, as well as age and body weight, showed substantial effects either on the efficiency of altrenogest or on reproduction parameters. It could be that the study population was too homogeneous with respect to body condition (and also “too” healthy) to see differences. Referring to the aforementioned study of Thitachot et al. [19], body condition, i. e., backfat thickness, has shown, in fact, to have effects, e.g., on the farrowing rate and on live born piglets.

While it was expected that prepubertal gilts would not perform as well as pubertal gilts with respect to reproductive performance [35,36,37,38], the results of this study failed to prove this concept. Conception and farrowing rates were similar, as were the numbers of total and life born piglets, as well as piglet birth weights. Prepubertal gilts are supposed to have a lower ovulation rate [39] and a lower uterine capacity [40] due, at least in part, to a less developed uterus than pubertal gilts [22,40,41], eventually leading to lower conception and lower litter size [35,36]. These relationships apparently did not apply to gilts of the breed used in this study (i.e., of Danish genetic stock). It might be assumed that the ovulation rate, generally known to be high in hyper-prolific breeds, is already high in first estrus gilts (i.e., determined as prepubertal at the beginning of altrenogest treatment in this study) to achieve high litter sizes [42]. Whether altrenogest, reported to increase ovulation rate [18,32,43,44], was beneficial to the prepubertal gilts remains unanswered, due to the objective and design of the presented study.

However, based on the observation of remarkably smaller uteri of prepubertal and pubertal gilts in this study, lower reproductive traits, especially towards a reduced litter size, would have been expected for prepubertal gilts. Why this was not observed in this study cannot be conclusively explained, but might be related to the parameter “cross-sectional area of the uterine horns”, which is not reliable (or feasible) for prediction of reproductive performance, as similarly previously observed by Lents et al. [45]. In their studies, the parameter ‘ultrasound measured diameter’ did not differ between the group directly selected for uterine capacity (which would increase the number of live born piglets) and the control group. Lents et al. [45] also did not find a strong correlation between the uterine horn diameter and length, as well as weight, thus length might be more crucial, to e.g., litter size than is diameter. However, using the entire data set, there was a slight but significant positive correlation between “cross-sectional area of the uterine horns” and live born piglets, and in tendency also for total piglets. This result may thus (re-)encourage the use of “cross-sectional area of the uterine horns” as a parameter when selecting for uterine capacity.

A few limitations should be considered when interpreting the results. The homogeneity of the gilt population, with similar age, body weight, and body condition, may have reduced variability and the ability to detect significant differences. Additionally, the lack of a control group (e.g., gilts of a different breed or a more heterogeneous group) limited comparative analysis. The relatively small sample size of the litters may have impacted the study. Future studies could address these limitations for more robust conclusions.

## 5. Conclusions

In conclusion, altrenogest (Altresyn ^®^) given orally at 20 mg/day/gilt reliably blocks follicular growth in hyper-prolific gilts of Danish genetic origin.

Under the conditions of this study (including optimal housing and management practices, a homogeneous gilt population and high reproductive performance), body condition, age and body weight, as well as pubertal status, did not have an effect on the efficacy of altrenogest or the reproductive outcome (except for uterine size, which was markedly smaller in prepubertal than in pubertal gilts and had a significant positive correlation with live born piglets). This may be partly due to the homogeneity of the gilt population, which may have limited the ability to detect variations in these factors. However, these findings also suggest that, under optimal farm management conditions, reproductive outcomes may not be strongly influenced by these variables. Future studies with more heterogeneous populations may help clarify the role of these factors in reproductive performance and the efficacy of altrenogest.

## Figures and Tables

**Table 1:** Descriptive statistics for the overall data set (n = 161 gilts; n = 70 litters for mean piglet weight).

Parameter	Gilts (n)	Value
Age beginning of altrenogest (days)	161	252.6 ± 7.5
Body weight beginning of altrenogest (kg)	161	153.0 ± 12.4
BFT beginning of altrenogest treatment (cm)	161	0.9 ± 0.2
BFT end of altrenogest treatment (cm)	161	1.1 ± 0.5
MT beginning of altrenogest treatment (cm)	161	6.0 ± 0.4
MT end of altrenogest treatment (cm)	161	6.2 ± 0.6
Follicle size end of altrenogest treatment (mm)	161	3.2 ± 0.4
Estrus rate post altrenogest (%)	160	99.4
Interval last altrenogest treatment-onset estrus (days)	160	5.9 ± 0.5
Conception rate (%)	160	100
Farrowing rate (%)	153	95.6
Total born piglets (n)	153	18.1 ± 2.6
Live born piglets (n)	153	17.4 ± 2.5
Stillborn piglets (n)	153	0.7 ± 1.0
Mean piglet weight (kg)	70	1.3 ± 0.2

BFT: Backfat thickness. MT: Muscle thickness.

**Table 2:** Distribution of gilts according to the interval between last altrenogest treatment and the onset of estrus (n = 160 in estrus).

Interval Last Altrenogest-Onset Estrus (Days)	5	5.5	6	6.5	7	8
Number animals n (%)	25 (15.6) ^b^	25 (15.6) ^b^	79 (49.4) ^a^	27 (16.9) ^b^	3 (1.9) ^c^	1 (0.6) ^c^

Values with different superscripts differ significantly (*p* ≤ 0.05).

**Table 3:** Correlation coefficient (*p*-value) for the relationship between data (body condition, age, body weight, and follicle size at the end of altrenogest treatment, as well as production traits; n = 161 gilts total; n = 70 litters for mean piglet weight).

Parameter.	Gilts/Litter (n)	BFT-1 (cm)	BFT-2 (cm)	MT-1 (cm)	MT-2 (cm)	Age (Days)	Body Weight (kg)
Follicle size end of altrenogest	161	0.11 (0.16)	0.15 (0.06)	−0.14 (0.07)	0.007 (0.92)	0.22 (0.004)	0.03 (0.66)
Interval last altrenogest treatment-onset estrus	160	−0.06 (0.47)	−0.02 (0.82)	0.19 (0.01)	0.08 (0.3)	−0.05 (0.53)	0.08 (0.31)
Conception rate	160	0.12 (0.12)	−0.14 (0.07)	−0.16 (0.04)	0.08 (0.33)	0.09 (0.27)	−0.09 (0.23)
Farrowing rate	153	0.12 (0.11)	−0.14 (0.07)	−0.16 (0.04)	0.08 (0.33)	0.09 (0.27)	−0.09 (0.23)
Total born piglets	153	0.04 (0.59)	0.09 (0.26)	0.06 (0.49)	0.05 (0.56)	−0.11 (0.16)	0.12 (0.13)
Live born piglets	153	0.04 (0.65)	0.06 (0.48)	0.02 (0.8)	0.05 (0.56)	−0.11 (0.18)	0.09 (0.28)
Stillborn piglets	153	0.02 (0.8)	0.1 (0.24)	0.1 (0.24)	0.007 (0.93)	−0.02 (0.75)	0.11 (0.2)
Mean piglet weight	70	−0.27 (0.02)	−0.15 (0.21)	0.05 (0.66)	0.26 (0.03)	−0.01 (0.93)	0.04 (0.73)

BFT-1: Backfat thickness beginning of altrenogest. BFT-2: Backfat thickness end of altrenogest. MT-1: Muscle thickness beginning of altrenogest. MT-2: Muscle thickness end of altrenogest.

**Table 4:** Comparison of data obtained for pubertal (n = 147) versus prepubertal gilts (n = 14).

Parameter	Pubertal		Prepubertal		*p*-Value
	n gilts	Value	n gilts	Value	
Age beginning of altrenogest (days)	147	252.8 ± 7.4	14	250.5 ± 8.7	0.65
Body weight beginning of altrenogest (kg)	147	152.6 ± 12.5	14	156.8 ± 10.8	0.28
BFT beginning of altrenogest (cm)	147	0.9 ± 0.2	14	0.8 ± 0.1	0.20
BFT end of altrenogest (cm)	147	1.0 ± 0.3	14	1.3 ± 1.5	0.12
MT beginning of altrenogest (cm)	147	6.0 ± 0.4	14	6.2 ± 0.3	0.04
MT end of altrenogest (cm)	147	6.2 ± 0.6	14	6.0 ± 0.6	0.29
Follicle size end of altrenogest (mm)	147	3.2 ± 0.4	14	3.0 ± 0.3	0.03
Estrus rate post altrenogest (%)	147	100	14	92.9	0.87
Interval last altrenogest treatment-onset estrus (days)	147	5.9 ± 0.5	14	5.7 ± 0.4	0.21
Conception rate (%)	147	100	13	100	N/A
Farrowing rate (%)	140	95.2	14	100	1.0
Total born piglets (n)	140	18.1 ± 2.6	13	17.6 ± 2.8	0.36
Live born piglets (n)	140	17.4 ± 2.5	13	17.1 ± 2.5	0.35
Stillborn piglets (n)	140	0.7 ± 1.0	13	0.5 ± 0.8	0.65
Mean piglet weight (kg)	65	1.3 ± 0.2	5	1.3 ± 0.2	0.44
Mean cross-sectional area of uterine horns (cm^2^)	132	1.63 ± 0.48	13	0.51 ± 0.15	<0.001

BFT: Backfat thickness. MT: Muscle thickness. N/A: Not applicable. The *p*-value could not be calculated due to the lack of variability, as both groups showed a 100% conception rate.

## Data Availability

All data supporting the findings of this study are included in this publication.
